# Effect of Cooking Method and Doneness Degree on Volatile Compounds and Taste Substance of Pingliang Red Beef

**DOI:** 10.3390/foods12030446

**Published:** 2023-01-18

**Authors:** Yujiao Wang, Xinrong Bu, Dawei Yang, Dan Deng, Zhaomin Lei, Zhaobin Guo, Xiaotong Ma, Li Zhang, Qunli Yu

**Affiliations:** 1 College of Food Science and Engineering, Gansu Agricultural University, Lanzhou 730070, China; 2 College of Animal Science and Technology, Gansu Agricultural University, Lanzhou 730070, China

**Keywords:** cooking method, doneness degree, Pingliang beef, volatile, umami

## Abstract

This study used gas chromatography–ion mobility spectrometry (GC-IMS) and high-performance liquid chromatography (HPLC) methods to examine the impact of cooking methods and doneness on volatile aroma compounds and non-volatile substances (fatty acids, nucleotides, and amino acids) in Pingliang red beef. The flavor substances’ topographic fingerprints were established, and 45 compounds were traced to 71 distinct signal peaks. Pingliang red beef’s fruity flavor was enhanced thanks to the increased concentration of hexanal, styrene, and 2-butanone that resulted from instant boiling. The levels of 3-methylbutanal, which contributes to the characteristic caramel–chocolate–cheese aroma, peaked at 90 min of boiling and 40 min of roasting. The FFA content was reduced by 28.34% and 27.42%, respectively, after the beef was roasted for 40 min and instantly boiled for 10 s (*p* > 0.05). The most distinctive feature after 30 min of boiling was the umami, as the highest levels of glutamate (Glu) (*p* < 0.05) and the highest equivalent umami concentration (EUC) values were obtained through this cooking method. Additionally, adenosine-5′-monophosphate (AMP) and inosine-5′-monophosphate (IMP) decreased with increasing doneness compared to higher doneness, indicating that lower doneness was favorable in enhancing the umami of the beef. In summary, different cooking methods and doneness levels can affect the flavor and taste of Pingliang red beef, but it is not suitable for high-doneness cooking.

## 1. Introduction

Flavor is the dominant factor in determining consumers’ decisions to purchase meat products, and they prefer to choose meat products that are tender and rich in flavor [1,2]. According to European consumer tests of European beef samples, flavor preferences accounted for the most significant proportion of palatability (39%), followed by tenderness (31%) and juiciness (24%) [3,4]. The cooking process has a significant impact on the flavor of meat products. The degree of cooking not only affects the flavor and consumer acceptance of meat products and their palatability [5,6] but also the cooking temperature, and time significantly affects the physicochemical properties and eating quality [7,8]. Meat undergoes a complex series of thermal reactions between the lean tissue and the non-volatile components of the fatty tissue at varying cooking times and temperatures, resulting in a wide variety of reaction products [9]. The Maillard reaction, caramelization, and lipid peroxidation are common explanations for these alterations [10,11]. Flavor precursors in meat, such as water-soluble amino acids, unsaturated fatty acids, reducing sugar, and intramuscular and subcutaneous fat, are mainly responsible for these changes [4,12,13,14].

When heated to the right temperature, the Maillard reaction or thermal degradation breaks down small molecules such as water-soluble amino acids, reducing sugars, ribose, and unsaturated fatty acids [9]. In contrast, the oxidative decomposition of fats breaks down larger fat molecules such as those found intramuscularly and subcutaneously [4,14], resulting in volatile flavoring substances such as heterocyclic compounds, aldehydes, ketones, esters, and acids [4,15]. Further decomposition of the unsaturated fatty acids yields volatile carbonyl compounds. Additionally, the hydrolysis of the hydroxy fatty acids yields acids, which, in turn, hydrolyze to give lactone compounds with meat aromas. Water-soluble amino acids react with reducing sugars during cooking, creating various volatile flavor substances such as pyrazine and pyridine [16]. Cerny et al. studied the aroma components of beef under various cooking conditions, and determined that 2-acetyl-2-thiazoline, furan mercaptan, 2-ethyl-3,5-dimethyl pyrazine, and 2,3-diethyl-5-methyl pyrazine are the primary flavor-active substances in roasted beef [17]. Gasser et al. discovered that the primary flavor-active compounds in boiled beef are aldehyde, 2-methyl-3-furan mercaptan, 2-acetyl-1-pyrroline, 2-formyl-5-methyl thiophene, trans-2,4-decadienal, and others [18], using solid-phase microextraction (SPME)–gas chromatography–mass spectrometry (GC/MS). Yunhe Zou et al. examined different ultrasound powers’ effects on five-spice beef volatile compounds. Different ultrasound intensities increased the volatile odors of cooked beef, as demonstrated by the results [19]. According to previous research, different cooking methods, temperatures, and times have significantly different effects on the flavor profile of beef.

However, most studies emphasize the changes in lipid oxidation and/or protein oxidation with temperature or time, and only a few replicate actual home cooking methods, such as roasting, barbecuing, and microwaving [20]. In addition, many authors only study the volatile compounds of cooked meat under different cooking processes, and the non-volatile compounds, such as inosine-5′-monophosphate (IMP), which typically have a significant impact on the umami flavor of meat, have been studied by only a small number of researchers. 5′-guanosine monophosphate (GMP) and amino acids, such as glutamate and aspartic acid, contribute significantly to umami flavor [21,22]. In addition, according to the United States Department of Agriculture Statistics (USDA), in 2020, the United States’ beef consumption was 12.531 million metric tons, ranking first in the world, accounting for 22.35% of the total global beef consumption; China and Brazil followed, with beef consumption of 9.486 million metric tons and 7.609 million metric tons, respectively, accounting for 16.92% and 13.57% of the total global beef consumption. Pingliang red beef is a Chinese yellow cattle breed, with fine, tender meat of exceptional quality and a distinctive flavor, suitable for roasted steaks or stir-frying [23,24]. In this study, HS-GC-IMS and HPLC were used to determine the volatile flavor compounds and non-volatile flavor substances (fatty acids, amino acids, and nucleotides) in the Pingliang red beef *longissimus dorsi* muscle under different cooking methods and different doneness to analyze the possible ways in which flavor and taste change.

## 2. Materials and Methods

### 2.1. Sample Preparation

Gansu Jingxing Beef Industry Co. was the supplier of the Pingliang red beef. The average age of the cattle was between 1.5 and 2 years, their average weight was approximately 330 kg, and all 9 originated from the same feeding lot. Pingliang beef cattle were fed a total mixed ration (TMR) consisting of alfalfa hay, straw, concentrates, corn silage, and grain mixtures, to meet or exceed their nutritional requirements as outlined by the National Research Council (NRC, 2003). Additionally, all cattle were fed twice daily at 07:00 and 15:00 with fresh feed. Upon completion of the 390-day finishing period, all cattle were slaughtered at a local slaughterhouse. The *longissimus dorsi* muscle was obtained immediately after humane slaughtering in compliance with the Operating procedure of livestock and poultry slaughtering—Cattle of the National Standards of P.R. China [25]. Before slaughter, all test cattle fasted for 24 h with a two-hour water restriction. Within 30 min of slaughter, the right carcass was cut vertically across the carcass from outside the 5th–6th lumbar vertebra to the middle of the 12th–13th thoracic vertebral fossa, and 4–5 kg of fresh boneless *longissimus dorsi* muscle was collected, followed by the removal and weighing of the subcutaneous fat. Before cooking and index analysis, the *longissimus dorsi* muscle was aged for 24 h in a refrigerator at 0 to 4 °C. The moisture content of Pingliang red beef was determined using the drying method, the ash content was determined using the high-temperature cautery method, the crude protein content was determined using the Kjeldahl method, and the fat content was determined using the Soxhlet extraction method, in which the moisture content was 73.11 g/100 g, ash content was 1.06 g/100 g, protein content was 21.79 g/100 g, and fat content was 3.35 g/100 g. In preparation for the cooking and processing part of the experiment, the collected beef was divided into four pieces weighing approximately 1.75 kg each, with the remaining beef used for raw meat analysis. After removal, all the meat samples were frozen at −20 °C for a maximum of 30 days, during which all the experiments in this study were conducted.

### 2.2. Different Cooking Methods of Pingliang Red Beef

**Figure 1:**
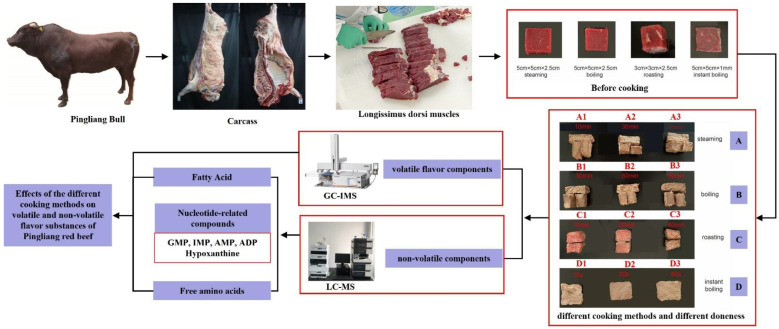
Experimental design of Pingliang red beef under different cooking methods.

Figure 1 depicts the experimental design of several cooking techniques for the *longissimus dorsi* muscles of Pingliang red beef. After removing the meat samples from freezing at −20 °C, they were placed in a clean container and defrosted at room temperature until the central temperature of the meat reached −5 °C–0 °C. After thawing, the fat, connective tissue, tendons, and muscle membranes on the surface of the meat samples were removed, and the meat was cooked using four different common home methods: steaming, boiling, roasting, and instant boiling. The beef samples were sliced into nine 5 cm × 5 cm × 2.5 cm pieces, soaked in a container for 15 min (water:meat mass ratio of 3:1), and taken out, and patted dry of surface water. Three pieces of meat were steamed in the steamer for 10 min, 30 min, and 60 min. Three parts were heated in a water bath pan, maintained at 90 °C for 30 min, 60 min, and 90 min, respectively. The remaining three pieces were oven-roasted. The oven temperature was adjusted to 160 °C for 10 min, 20 min, and 40 min, respectively. The samples were flipped once every 5 min to provide even heating. After cooking, each beef sample was chopped and frozen at −20 °C. The *longissimus dorsi* muscle was sliced using a slicer into slices 2 mm thick (5 cm × 5 cm) before being cooked in an induction cooker. After boiling, the power was reduced to 1000 W, and the cooking times were 10 s, 30 s, and 60 s, respectively. According to Table 1, the experimentally treated samples were numbered as indicated. No seasonings were included. After cooking, the meat was chopped and frozen at −20 °C.

### 2.3. HS-GC-IMS Analysis

Before the experiment, the samples were retrieved from the −20 °C freezer and thawed at room temperature until the central temperature of the sample reached −5 °C–0 °C. Three grams of each sample were weighed and transferred to empty 20 mL vials with magnetic screw caps, and the headspace samples were incubated. The samples in the headspace vials were incubated, and the headspace components were extracted using a hot injector and examined using a gas chromatography–ion mobility spectrometer (GC-IMS) (Flavourspec, G.A.S. Instrument, Shandong Haineng Scientific Instruments Co., Ltd., Shanghai, China) for volatile components. Table 2 lists the criteria for chromatographic analysis. Each sample was obtained as a parallel sample three times [1].

### 2.4. Fatty Acid Analysis

Fatty acid analysis was conducted following the method of Xiao et al. with slight modification [26]. A total of 50 mg of freeze-dried beef sample was accurately weighed and placed in a 2 mL glass centrifuge tube. A total of 2 mL of 5% *v*/*v* sulfuric acid in methanol was added, followed by 100 μL of 1 mg/mL undecanoic acid triglyceride (C11:0) in methanol as an internal standard. After that, 25 Μl BHT methanol solutions (0.2%, *m*/*v*) were added to a headspace sample vial. The enclosed mixture was heated for 1.5 h at 90 °C after vortexing for 10 s. At the end of the reaction, the bottle was opened when the temperature reached room temperature. The 2 mL saturated sodium chloride solution was added followed by 4 mL of hexane. The vial was vortexed for 30 s. After standing for 15 min, 1 mL supernatant was transferred into a 1.5 mL centrifuge tube. The supernatant was centrifuged at 15,700× *g* for 2 min at 20 °C and 200 μL of the supernatants were used for GC-MS analysis. For chromatographic examination, the extract was filtered through a 0.22 µm high-performance liquid chromatography flask (Agilent, Santa Clara, CA, USA). The following are the chromatographic analysis and measurement parameters: The chromatographic column was an HP-5MS capillary column (30 m × 0.25 mm × 0.25 µm film thickness, Agilent, USA); the column temperature was 150 °C (for a duration of 3 min), which was increased to 180 °C at a rate of 2.5 °C/min, and then, sustained for 5 min. The temperature was then increased to 220 °C at a rate of 2.5 °C/min while the carrier gas flow rate was maintained at 0.7 mL/min for 25 min. The fatty acid composition was identified using a mass spectrometry database (NIST Library, Mass spectrometry Retrieval Program, version 5.0, USA).

### 2.5. Nucleotide-Related Compound Determination

High-performance liquid chromatography (HPLC) was used to analyze adenosine-5′-diphosphate (ADP), inosine-5′-monophosphate (IMP), 5′-guanosine monophosphate (GMP), adenosine-5′-monophosphate (AMP), and hypoxanthine. According to the modified method of Jayasena et al. [27]. Beef samples (5 g) were minced, followed by the addition of 20 mL of cold 0.5 mol/L perchloric acid and homogenization at 12,000× *g* for 15 s. The solution was centrifuged 4 times at 3000× *g* for 10 min. The supernatant was filtered using filter paper at medium speed. The precipitate was then combined with 10 mL of pre-chilled 0.5 M perchloric acid and the supernatant was combined in a 50 mL conical bottle. We adjusted the pH and correctly measured 20 mL of the extract before transferring it to a 100 mL volumetric flask, filtering it through a 0.22 µm filter membrane into HPLC vials (Agilent, Santa Clara, CA), and then, waiting for the machine to perform the measurement.

HPLC [21] was performed on a TC-C18 column with a detection wavelength of 254 nm and a column temperature of 25 °C. A 95% phosphate buffer solution (7.0) and 5% methanol solution (HPLC grade) were used for gradient injection at a flow rate of 0.8 mL/min, and the injection volume was 10µL. The gradient elution settings were: 0–10 min, 99% phosphate buffer solution (7.0) and 1% methanol; 12 min, 80% phosphate buffer solution (7.0); and 20% methanol for 6 min. The phosphate buffer solution increased to 99% within 2 min and remained for another 8 min. At the end of each injection, a mixture of water and methanol (1:1 *v*/*v*) was used for purification. The standard external method for quantitative analysis was used to prepare the mixed standard solution of IMP AMP, hypoxanthine, ADP, and GMP. Each substance’s gradient of mass concentration was 1, 2.5, 5, 10, 20, 50, and 100 mg/L. When the same sample was subjected to the presentation of various mass concentrations of flavor nucleotide standard solution, the chromatogram peak range of mass concentration of the figure was obtained. The standard curve of the five different types of nucleotides was then compared to the standard retention time to identify the nucleotides in the samples, according to the calibration curve, and provides quantifiable data on the sample in mg/100 g^−1^.

### 2.6. Equivalent Umami Concentration (EUC)

Determination and analysis of non-volatile flavoring agents: Monosodium glutamate equivalent (EUC) (g MSG/100 g) is the monosodium glutamate concentration (MSG, g/100 g), which is composed of the umami amino acid (Asp or Glu) and the 5′-nucleotide (IMP, AMP, and GMP) and synergistically represents the contribution to the umami value of food [28]: The calculation formula is as follows:

$$\mathrm{EUC}=\sum \alpha_{1}\beta_{1}+1218\left(\sum \alpha_{1}\beta_{1}\right)\left(\sum \alpha_{2}\beta_{2}\right)$$

In the formula, EUC equals the concentration of sodium glutamate (g MSG/100 g). The value of 1218 is the coefficient of synergy; α_1_ refers to the concentration of Asp Glu (g/100 g); β_1_ refers to the relative freshness coefficient of the Asp Glu equivalent to MSG (Asp:0.077, Glu:1); α_2_ refers to the 5′-GMP, 5′-AMP, and 5′-IMP concentration (g/100 g); and β_2_ refers to the 5′-GMP, 5′-AMP, and 5′-IMP relative to the improvement of the relative coefficient of 5′-IMP (5′-AMP: 0.18, 5′-IMP: 1, 5′-GMP: 2.3).

### 2.7. Free Amino Acid Determination

Pretreatment of beef samples was performed according to Nahar Sabikun et al. [29] with minor modifications. First, 250 mg of the freeze-dried beef samples was weighed and placed in a hydrolysis tube 16 h after hydrolysis. Then, 1 mL was placed in a corked test tube and oven-dried for 4 h (the oven door was slightly open throughout the process). The dried precipitate was dissolved in ultrapure water, and the appropriate amount of dissolved solution was filtered into a high-performance liquid chromatography bottle (Angelon, Santa Clara, CA) with a 0.22 µm filter membrane for analysis. FAA was evaluated using an automated amino acid analyzer (L-8900; Hitachi, Tokyo, Japan) to determine the free amino acid content, per Jun Qi et al. [28]. The temperature of the reactor was 135 °C, and the temperature of the column was 50 °C. Three times, the absorbance at 440 nm and 570 nm were measured.

### 2.8. Statistical Analysis

The findings are reported as the mean ± standard deviation for all tests, which were conducted in triplicate. The heat map was analyzed using R programming language software (version 3.6.1). The GC-IMS library assessed characteristic aroma components using LAV (Laboratory Analytical Viewer) and the NIST2014 and IMS databases. The Reporter plugin of LAV was used to directly examine spectrum discrepancies between samples, while the Gallery plot plugin was utilized to visually and quantitatively compare changes in volatile chemicals’ different cooking methods. The experimental data are reported as the mean ± standard error. The data were statistically analyzed using SPSS 26.0 (SPSS Institute Ltd., Somers, NY, USA), and multiple comparisons of treatments were performed using Duncan’s multiple-range test.

## 3. Results and Discussion

### 3.1. GC-IMS Fingerprint Analysis of Pingliang Red Beef under Different Cooking Methods

**Figure 2:**
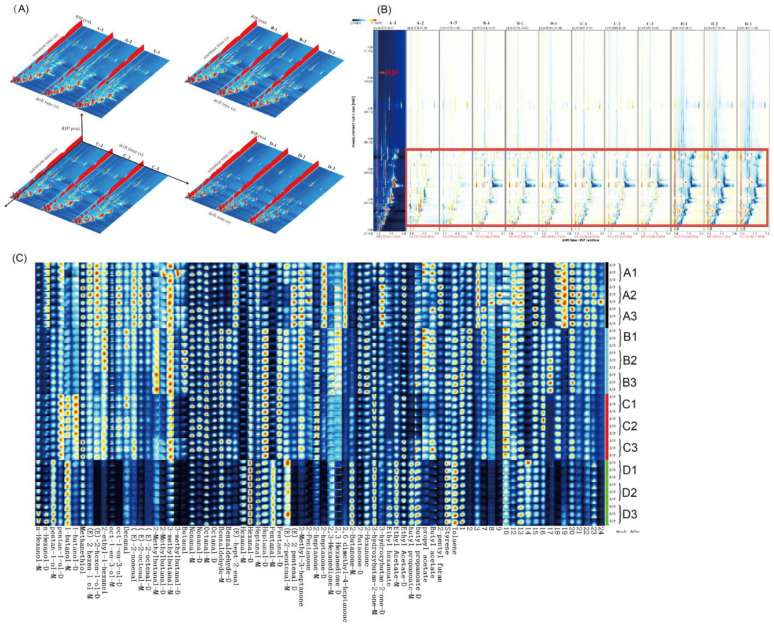
(**A**) Three-dimensional topographic plots of Pingliang red beef samples from different cooking samples. (**B**) Two-dimensional topographic plots of Pingliang red beef samples from different cooking samples. (**C**) GC-IMS fingerprints of Pingliang red beef samples from different cooking samples (In the figure, A stands for steaming, where A1 stands for steaming for 10 min, A2 stands for steaming for 30 min, and A3 stands for steaming for 60 min. B stands for boiling, where B1 stands for boiling for 30 min, B2 for 60 min, and B3 for 90 min. C means roasting, where C1 means roasting for 10 min, C2 means roasting for 20 min, and C3 means roasting for 40 min. D indicates instant boiling. D1 indicates instant boiling for 10 s, D2 indicates instant boiling for 30 s, and D3 indicates instant boiling for 60 s).

GC-IMS was used to analyze the flavor of the volatiles in different cooking methods and at different doneness. Figure 2A is a three-dimensional representation of the data, with the entire spectrum indicating all volatile components in the sample’s headspace. As seen in the 3D plot, the volatile components of Pingliang red beef are very similar under different cooking methods; however, the signal intensities are slightly different, and the signal intensities do not vary significantly for different cooking doneness of the same cooking method (Figure 2A). In addition, the area of blue spots in the instant boiling group (D-1, D-2, and D-3) is significantly larger than in the other three cooking methods, indicating that the instant boiling group contains fewer volatile compounds than the other three cooking methods (steaming, boiling, and roasting) (Figure 2B). As depicted in Figure 2B, the area of the red spots of volatile compounds in Pingliang red beef reduces considerably with increasing steaming doneness (A-2 and A-3) compared to steam for 10 min (A-1) in the 100–300 s retention time range. This could be because an increase in temperature favors lipid oxidation and promotes the release or concentration of specific components. Some volatile compounds are temperature-sensitive and easily synthesized or degraded, resulting in the disappearance or reduction of specific signals after 60 min of steaming.

The topographical map can immediately show the changing trend of total volatile components in red Pingliang beef under different cooking methods; however, it is difficult to assess the changes in each volatile component accurately. The GalleryPlot plugin of the software LAV was used to select all peaks to be studied in the two-dimensional spectrum of GC-IMS for a more exhaustive comparison of the variations in volatile components between samples of Pingliang red beef cooked using various techniques. This process was repeated three times for every sample (Figure 2C). The Y-axis on the right in Figure 2C represents the number of samples, while the X-axis represents the signal peaks of all volatiles picked from the beef samples, with qualitative compounds represented by their names and non-qualitative compounds represented by their quantities. In twelve groups of red beef, all characteristic peak intensities were identified, and differences in characteristic odors were evaluated (Figure 2C). From the whole fingerprint, it can be seen that Pingliang red beef samples with 12 groups of different cooking methods have common flavor regions and characteristic peak regions. The qualitative analysis depicted in Figure 2C reveals that the volatile components detected in Pingliang red beef prepared using four distinct cooking methods and varying degrees of doneness consist of 71 types of monomers and a portion of dimers. A total of 16 aldehydes, 10 ketones, 9 alcohols, 6 esters, 2 heterocycles, 1 furan, and 1 acid make up most of the monomers. The volatile component concentrations of the four distinct cooking procedures differ significantly, with the greatest differences occurring between instant boiling and the other three cooking methods (Figure 2C). Heptanal, an aldehyde with a fatty flavor, has a much higher signal intensity under boiling and roasting than steaming and instant boiling; hexanal, an aldehyde with a fruity flavor, has the maximum signal intensity under instant boiling; and heptanal, pentan-1-ol, 1-butanol, and 3-methylbutanal have the strongest signals during roasting. Heptanal, 2-ethyl-1-hexanol, 2-Methylbutanol, and 3-methylbutanal have the strongest signals during cooking; 3-methylbutanal increases with increasing boiling and roasting doneness, resulting in stronger odors of chocolate and cheese at 90 min of boiling and 40 min of roasting. Under 30 min of steaming, the signal strength of (E)-2-octenal, (E)-2-nonenal, 2-methyl-3-heptanone, and 2-pentylfuran increase, indicating that the fatty odor of Pingliang red beef is more pronounced.

### 3.2. Volatile Compound Variation in Steaming, Roasting, Boiling, and Instant Boiling of Pingliang Red Beef

**Figure 3:**
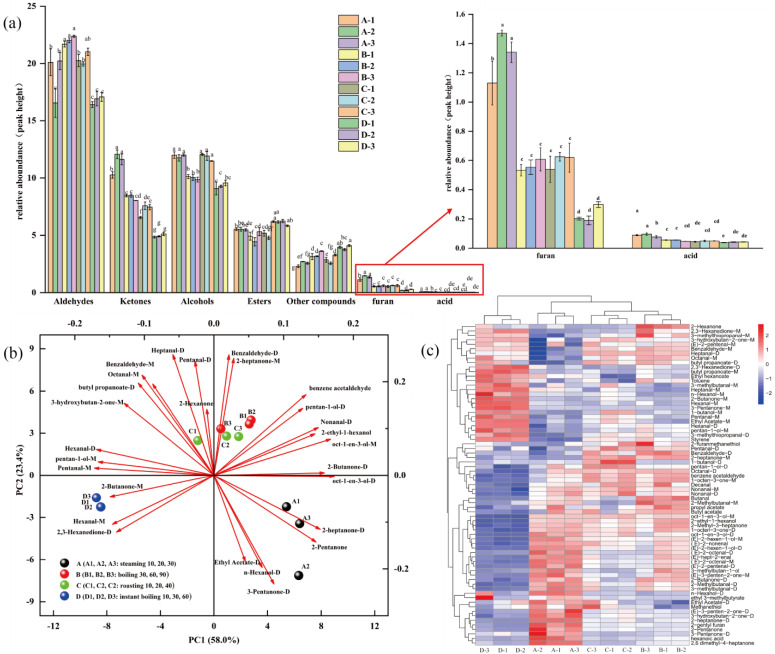
(**a**) Changes in the abundance of volatile components in Pingliang red beef under 12 different cooking methods. Different letters (a–g) in bars for different samples indicate significant differences (*p* < 0.05). (**b**) Principal component analysis of Pingliang red beef samples from different cooking samples. (**c**) Heat map visualization and clustering results of the volatile compounds in beef samples.

Table 3 lists all 71 detected chemicals (monomers and dimers), including aldehydes, ketones, alcohols, esters, furans, acids, and heterocyclic compounds, as determined by the additional qualitative investigation. According to Table 3 and Figure 3a, aldehydes were the most prevalent and had the highest concentration among the volatile chemicals discovered using the various cooking methods, followed by alcohols and ketones, with acids and furans being the least prevalent. To show the changes in the volatile components of Pingliang red beef under different cooking methods and doneness, signal intensity was used to highlight the differences in volatile components via PCA (Figure 3b), and a heat map of the volatile components was generated in R (Figure 3c). Principal component analysis aims to convert multiple indices into multiple comprehensive indices by reducing the dimension, extracting multiple principal components from the original variables, approximating the original variables, and preserving as much information as possible from the original variables. The better the correlation between the original variables, the fewer new variables are required and the greater their dissimilarity [30]. A. B, C, and D represent the changes in the major constituents of Pingliang red beef during steaming, boiling, roasting, and instant boiling, respectively. Due to the large sample size, the contribution of PC1 and PC2 explains more than 77.3% of the total variation, and the four groups of different cooking methods can be distinguished, indicating that the flavor profiles of the samples differ. The closer the samples are to one another in the graph, the greater their resemblance and relative taste-component content. The dimensionality of reduced data can adequately characterize the original data, and it is possible to observe the characteristic distinctions between samples. The general characteristics of the two cooking methods of boiling and roasting are closer to the middle of Figure 3b, and the principal components are fairly comparable. The left and right sides of the diagram illustrate the clear distinction between steaming and quick cooking. The repeated samples in the graph may be grouped closely, and for the same cooking process, there is essentially no difference between the flavors at different maturities. Cluster analysis of the heat map reveals similarities and differences across various treatments, which is congruent with the results of principal component analysis. As indicated in Figure 3c, the levels of volatile compounds in Pingliang red beef vary significantly with the degree of cooking (roasting, boiling, and instant boiling), with the largest content of propyl acetate occurring when the beef is steamed for 10 min or boiled for 30 min. This indicates that propyl acetate decomposes with increasing heating duration, and the concentration of propyl acetate falls with increasing steaming and boiling time. The higher the concentration of 3-hydroxybutan-2-one, the stronger the cream flavor of Pingliang red beef at steaming times of 30 and 60 min, and the higher the concentration of (E)-2-pentenal-M, the longer the steaming time. The various cooking techniques have a higher impact on the flavor of Pingliang red beef than the degree of maturity, which the various heat transfer techniques should primarily influence.

Aldehydes have a low threshold and have primarily meat-based fatty aromas. Aldehydes are comparatively volatile because of the oxidation of unsaturated fatty acids [31,32]. In addition, the Strecker amino acid reaction and the decomposition of carbohydrates can produce certain aldehyde molecules [14,30,33,34]. Table 3 and Figure 3a show that different cooking methods of Pingliang red beef produce aldehyde compounds at the highest levels in volatile compound types; therefore, their contribution to the overall aroma of volatile aldehydes is likely the largest. These results are consistent with the research results of Dominguez et al., 2014, namely that in terms of volatile compounds, all cooked beef samples had the highest levels of aldehydes [35,36,37]. In this experiment, the following aldehydes were identified: decanal, (E)-2-nonenal, nonanal, (E)-2-octenal, octanal, benzaldehyde, benzeneacetaldehyde, (E)-hept-2-enal, hexanal, heptanal, 2-methylbutanal, 3-methylthiopropanal, pentanal, butanal, (E)-2-pentenal, and 3-methylbutanal, but the boiling procedure produced the highest aldehyde concentration, followed by roasting and steaming for 10 and 60 min, respectively. The Pingliang red beef fat flavor was the most prominent among the four cooking methods. Figure 2c shows that instant-boiled Pingliang red beef is rich in hexaldehyde and heptaldehyde. Hexanal and heptanal are oxidation products of linoleic acid and arachidonic acid [38], with green, fresh sebaceous, and fatty tastes. The instant boiling under the flat coolness of Pingliang red beef makes the umami flavor more impressive. The concentration of nonanal and octanal in Pingliang red beef after 20 min of roasting is much higher than after 10 and 40 min. Nonanal and octanal are generated through the oxidation of oleic acid [39,40] with a roasted flavor, indicating that Pingliang red beef cooked for 20 min has the strongest barbecue flavor. The concentration of benzaldehyde after 30 min of steaming is much greater than after 10 min and 60 min of steaming. Benzaldehyde is a phenylalanine breakdown product [41] with an almond, burnt sugar, and sweet flavor. After 30 min of steaming, the Pingliang red beef tastes more like burnt sugar than after 10 min and 60 min.

Alcohols have less evident effects on the flavor of meat than aldehydes, but they play a crucial part in creating the overall flavor of the meat. Saturated alcohols, mostly generated via lipid oxidation and the Strecker degradation reaction, has a higher flavor threshold and contributes less to beef jerky’s overall flavor than unsaturated alcohols, which have a lower flavor threshold and contribute more to the flavor [42]. 2-ethyl-1-hexanol, oct-1-en-3-ol, n-hexanol, (E)-2-hexen-1-ol, pentane-1-ol, 3-methyl butan-1-ol, 3-hydroxybutan-2-one, 1-butanol, and methanethiol were the alcohols identified in this study. When cooked to medium doneness, oct-1-en-3-ol concentration is at maximum compared to the other cooking methods. The concentrations of 2-ethyl-1-hexanol are substantially higher in steaming, boiling, and roasting than in instant boiling. Steaming contains substantially more (E)-2-hexen-1-ol than the other cooking methods, resulting in a flavor that is more bell pepper, fat, and green. Only after 30 min of steaming does the largest concentration occur of 3-hydroxybutan-2-one, which imparts a buttery, creamy flavor. Methanethiol is at maximum only when roasted for 40 min, possibly because the thermal degradation of thiamine produces more sulfur-containing compounds as the oven temperature increases [43], thus contributing to the production of the meat’s essential flavor. The 3-methyl butan-1-ol content and fruit flavor are greatest after 10 min of roasting; as the roasting time increases, the 1-butanol content decreases, and the fruit flavor diminishes.

2-methyl-3-heptanone, 1-octen-3-one, 2-pentanone, 2-butanone, 2-heptanone, 2,3-hexanedione, (E)-3-pentene-2-one, 2-hexanone, 2,6-dimethyl-4-heptanone, and 3-pentanone were identified as ketones in this experiment. In addition to thermal oxidation or the decomposition of unsaturated fatty acids and amino acids [14,31,44], the formation of ketone molecules in processed beef also involves the decomposition of unsaturated fatty acids and amino acids. However, most ketones have a high threshold value and contribute little to flavor characteristics, although certain are crucial intermediates in the production of heterocyclic compounds and play a substantial role in the formation of meat flavor [45]. The increased levels of 1-octen-3-one, 2-methyl-3-heptanone, and 2-heptanone after steaming and boiling compared to roasting and instant boiling gave Pingliang red beef a boiled mushroom flavor. The concentration of 2-hexanone and 2,3-hexanedione increase as boiling time increases.

In this experiment, esters were less detectable, their volatility was low, and their effect on the overall flavor was small. In this experiment, six esters were identified, three of which had a fruity flavor: ethyl hexanoate, ethyl acetate, and butyl acetate. The concentrations of ethyl acetate, an odorless, fruity liquid, are much greater during steaming and instant boiling than during boiling and roasting. With increasing roasting time, the amount of butyl acetate gradually reduces, indicating that the fruity flavor of Pingliang red beef diminishes. The concentration of 2-pentylfuran is highest during the steaming process, and it steadily increases as cooking time increases, indicating that the fruit flavor becomes stronger as the steaming duration grows [46].

**Table 3:** Qualitative information on samples of Pingliang red beef processed using four cooking methods.

	Compound	CAS	Formula	MW	RI (s)	Dt	Sensory Descriptions [46]
Aldehydes	Decanal	C112312	C_10_H_20_O	156.3	1277.1	1.53797	Fresh grease, fruity, soapy
	(E)-2-nonenal	C18829566	C_9_H_16_O	140.2	1188.1	1.4107	Intense floral, fruity and oily aromas, soapy
	Nonanal-M	C124196	C_9_H_18_O	142.2	1109.1	1.47344	Floral, aldehyde-like, citrus, soapy, fried fragrant, roasted fragrant
	Nonanal-D	C124196	C_9_H_18_O	142.2	1108.1	1.94666	
	(E)-2-octenal-M	C2548870	C_8_H_14_O	126.2	1054.7	1.33541	Meaty, nutty, grease, cucumber flavor, umami
	(E)-2-octenal-D	C2548870	C_8_H_14_O	126.2	1054.7	1.82835	
	Octanal-M	C124130	C_8_H_16_O	128.2	1008.6	1.40712	Grease, citrus, soapy
	Octanal-D	C124130	C_8_H_16_O	128.2	1004.5	1.82656	
	Benzaldehyde-M	C100527	C_7_H_6_O	106.1	956.7	1.15172	Almond, burnt sugar, sweet
	Benzaldehyde-D	C100527	C_7_H_6_O	106.1	957.1	1.47171	
	benzene acetaldehyde	C122781	C_8_H_8_O	120.2	1039.3	1.25813	Sweet, intense floral
	(E)-hept-2-enal	C18829555	C_7_H_12_O	112.2	952.3	1.67411	
	Hexanal-M	C66251	C_6_H_12_O	100.2	791.9	1.25392	Grassy, green, fresh, tallow, fat
	Hexanal-D	C66251	C_6_H_12_O	100.2	787.2	1.56558	
	Heptanal-M	C111717	C_7_H_14_O	114.2	898.2	1.32579	Grassy, green, fat,
	Heptanal-D	C111717	C_7_H_14_O	114.2	898.9	1.69956	
	2-Methylbutanal-D	C96173	C_5_H_10_O	86.1	654.4	1.40175	Almond, chocolate, cocoa, fermented, hazelnut
	2-Methylbutanal-M	C96173	C_5_H_10_O	86.1	652.8	1.15776	
	3-methylthiopropanal-M	C3268493	C_4_H_8_OS	104.2	905.2	1.08897	
	3-methylthiopropanal-D	C3268493	C_4_H_8_OS	104.2	904.7	1.40189	
	Pentanal-M	C110623	C_5_H_10_O	86.1	693.4	1.18321	Almond, chemical, green, malt, oil
	Pentanal-D	C110623	C_5_H_10_O	86.1	692.1	1.43008	
	Butanal	C123728	C_4_H_8_O	72.1	540.3	1.28464	Banana, green, pungent
	(E)-2-pentenal-D	C1576870	C_5_H_8_O	84.1	745.3	1.35813	apple, floral, green, strawberry, tomato
	(E)-2-pentenal-M	C1576870	C_5_H_8_O	84.1	744.8	1.10603	
	3-methylbutanal-D	C590863	C_5_H_10_O	86.1	638.4	1.41016	Almond, cheese, chocolate, malt
	3-methylbutanal-M	C590863	C_5_H_10_O	86.1	635.1	1.17318	
Ketones	2-Methyl-3-heptanone	C13019200	C_8_H_16_O	128.2	1085.8	1.27626	
	1-octen-3-one-M	C4312996	C_8_H_14_O	126.2	979.4	1.27092	Boiled mushroom, earth, green, metal, mushroom
	1-octen-3-one-D	C4312996	C_8_H_14_O	126.2	978.7	1.68592	
	2-Pentanone	C107879	C_5_H_10_O	86.1	676.5	1.37584	Burnt plastic, ether, fruit, kerosine, orange peel
	2-Butanone-D	C78933	C_4_H_8_O	72.1	566.8	1.24553	Butterscotch, ether, fragrant, fruit, pleasant
	2-Butanone-M	C78933	C_4_H_8_O	72.1	565.1	1.06071	
	2-heptanone-M	C110430	C_7_H_14_O	114.2	891.8	1.25627	Bell pepper, blue cheese, cinnamon, green, nut
	2-heptanone-D	C110430	C_7_H_14_O	114.2	890.1	1.63201	
	2,3-Hexanedione-M	C3848246	C_6_H_10_O_2_	114.1	778.3	1.09198	
	2,3-Hexanedione-D	C3848246	C_6_H_10_O_2_	114.1	772.7	1.35966	
	(E)-3-penten-2-one-D	C3102338	C_5_H_8_O	84.1	731.8	1.35105	
	(E)-3-penten-2-one-M	C3102338	C_5_H_8_O	84.1	734.2	1.09263	
	2-Hexanone	C591786	C_6_H_12_O	100.2	779.5	1.18752	Ether
	2,6-dimethyl-4-heptanone	C108838	C_9_H_18_O	142.2	967.8	1.32856	
	3-Pentanone-D	C96220	C_5_H_10_O	86.1	692.6	1.35946	Ether, sweet
	3-Pentanone-M	C96220	C_5_H_10_O	86.1	693.2	1.10836	
Alcohols	2-ethyl-1-hexanol	C104767	C_8_H_18_O	130.2	1026.9	1.26013	Citrus, green, oil, rose
	oct-1-en-3-ol-M	C3391864	C_8_H_16_O	128.2	986	1.15975	earth, fat, floral, green, herb
	oct-1-en-3-ol-D	C3391864	C_8_H_16_O	128.2	979.4	1.60406	
	n-Hexanol-M	C111273	C_6_H_14_O	102.2	861.9	1.32497	Bread, flower, fruit, green, herb
	n-Hexanol-D	C111273	C_6_H_14_O	102.2	861.9	1.6385	
	(E)-2-hexen-1-ol-M	C928950	C_6_H_12_O	100.2	846	1.18518	Bell pepper, fat, geranium, green, spice
	(E)-2-hexen-1-ol-D	C928950	C_6_H_12_O	100.2	844	1.51752	
	pentan-1-ol-M	C71410	C_5_H_12_O	88.1	762.4	1.25392	Balsamic, fruit, green, medicine, yeast
	pentan-1-ol-D	C71410	C_5_H_12_O	88.1	764.4	1.51286	
	3-methylbutan-1-ol	C123513	C_5_H_12_O	88.1	727.3	1.48806	Banana, cocoa, floral, fruit, fusel
	3-hydroxybutan-2-one-M	C513860	C_4_H_8_O_2_	88.1	712.8	1.05627	Butter, cream
	3-hydroxybutan-2-one-D	C513860	C_4_H_8_O_2_	88.1	713.2	1.33282	
	1-butanol-M	C71363	C_4_H_10_O	74.1	654.3	1.1823	Alcohol, fruit, medicine
	1-butanol-D	C71363	C_4_H_10_O	74.1	653	1.37683	
	Methanethiol	C74931	CH_4_S	48.1	441.9	1.0483	Cabbage, garlic, gasoline, putrid, sulfur
	2-furanmethanethiol	C98022	C_5_H_6_OS	114.2	912.8	1.11099	Burnt, coffee, green, metal, roast
Esters	Ethyl hexanoate	C123660	C_8_H_16_O_2_	144.2	999.5	1.34258	Apple peel, banana, brandy, cheese, overripe fruit
	Ethyl Acetate-D	C141786	C_4_H_8_O_2_	88.1	601.1	1.34402	Balsamic, fruit, grape, pineapple
	Ethyl Acetate-M	C141786	C_4_H_8_O_2_	88.1	599.1	1.0987	
	butyl propanoate-M	C590012	C_7_H_14_O_2_	130.2	907.3	1.28334	Red fruit, strawberry
	butyl propanoate-D	C590012	C_7_H_14_O_2_	130.2	907.1	1.72743	
	Ethyl 3-methylbutyrate	C108645	C_7_H_14_O_2_	130.2	842.7	1.65769	Apple, citrus, pineapple, sour, sweet
	propyl acetate	C109604	C_5_H_10_O_2_	102.1	703.6	1.47843	Celery, floral, pear, red fruit
	Butyl acetate	C123864	C_6_H_12_O_2_	116.2	816.1	1.61605	Apple, banana, pungent, sweet
Furan	2-pentylfuran	C3777693	C_9_H_14_O	138.2	994.1	1.2552	Floral, fruit, green, green bean
acid	hexanoic acid	C142621	C_6_H_12_O_2_	116.2	994.1	1.30503	Acid, cheese, goat, pungent, rancid
Hydrocarbons	Styrene	C100425	C_8_H_8_	104.2	887.9	1.41987	Balsamic, gasoline, plastic, rubber, solvent
	Toluene	C108883	C_7_H_8_	92.1	760.5	1.01492	Bitter almond, glue, paint, solvent

Abbreviations: DT: drift time; MW: molecular weight; RI: retention index.

### 3.3. Nonvolatile Compounds

#### 3.3.1. Fatty Acid

**Figure 4:**
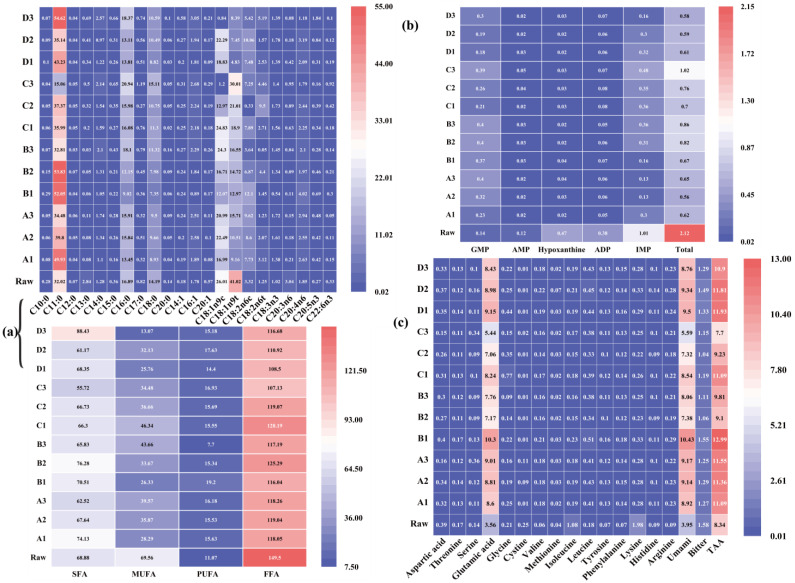
(**a**) Heat map visualization and clustering results of the fatty acid in beef samples (FFA: total free fatty acids; PUFA: polyunsaturated fatty acid; MUFA: monounsaturated fatty acid; SFA: saturated fatty acid); (**b**) heat map visualization and clustering results of the nucleotides in beef samples; (**c**) heat map visualization and clustering results of the free amino acids in beef samples. (Umami: sum of aspartic and glutamic acid. Bitter: sum of histidine, arginine, valine, methionine, phenylalanine, isoleucine, and leucine.)

Free fatty acids are important flavor precursors in meat products. They are liberated by lipid hydrolysis during meat processing [47], where the main volatile flavor compounds (aldehydes, ketones, and alcohols) during processing are primarily derived from the oxidative degradation of fatty acids [47,48]. The majority of the aldehydes, such as hexanal and heptanal, in Pingliang red beef are derived from the oxidation of unsaturated fatty acids, particularly polyunsaturated fatty acids (PUFA), which are more easily oxidized in their free state than in their bound state [47,49,50]. Unsaturated fatty acids have a grassy odor and are the primary byproduct of omega-6 fatty acid peroxide breakdown [31]. As depicted in Figure 4 and Table 4, a total of 22 fatty acids were identified in the *longissimus dorsi* muscle of raw and cooked Pingliang red beef, including 10 saturated fatty acids and 12 unsaturated fatty acids (5 monounsaturated fatty acids and 7 polyunsaturated fatty acids). As shown in Figure 4 and Table 4, the total fatty acid content in raw Pingliang red beef is significantly higher than that after cooking, indicating that several fatty acid interactions occur during the cooking process, such as lipid oxidation reactions occurring primarily during the steaming, boiling, and instant boiling processes and Maillard reactions occurring primarily during the roasting process [51]. A two-factor interaction analysis revealed that saturated fatty acid (SFA) and monounsaturated fatty acid (MUFA) show extremely significant differences (*p* < 0.01) and total free fatty acids (FFAs) show significant differences among the four cooking methods of Pingliang red beef. Pingliang red beef shows significant differences (*p* < 0.05) in SFA, MUFA, PUFA, and FFAs when cooked to different degrees of doneness, and the interaction effect of different cooking methods and different doneness degrees is extremely significant (*p* < 0.01). Total free fatty acid content decreases by 28.34% and 27.42% (*p* < 0.05) in the roasted (40 min) and instant boiling (10 s) groups, respectively, compared to the raw meat group (Table 4). The PUFA and SFA contents decrease gradually with doneness in the steamed and boiled groups, but the opposite pattern is observed in the instant group (Figure 4). It is assumed that the content of PUFA and SFA is related not only to the cooking method and doneness but also to the thickness of the meat during the cooking process. Beef is processed to yield a variety of volatile chemicals, including octanal, decanal, nonanal, hexanal, 1-heptanol, 1-octen-3-ol, 1-penten-3-ol, 3-octanone, and 2-nonanone, primarily via autoxidation/enzymatic oxidation [52,53]. Among the 12 different cooking processes, oleic (C18:1n9c) and monounsaturated fatty acids have the highest total content in the boiled (90 min) and roasted (10 min) groups, as monounsaturated fatty acids such as oleic acid can be cleaved to form aldehydes. This is similar to the results of the IMS study, where the aldehyde content was higher in the boiled (90 min) and roasted (10 min) groups of Pingliang red beef [52,54]. Linoleic, linolenic, arachidonic, eicosapentaenoic, and docosahexaenoic acids can be cleaved to generate aromatic chemicals [55], such as benzaldehyde and styrene. Table 4 and Figure 4 demonstrate that the levels of linoleic acid (C18:2n6c) and PUFA are considerably greater in the boiling (30 min) and instant boiling (30 s) groups than in the other groups. In accordance with the IMS findings, benzaldehyde and styrene are more prevalent in these two cooking procedures than in the others.

#### 3.3.2. Nucleotide-Related Compounds

Flavor nucleotides are significant flavor molecules in meat. Figure 4 and Table 4 illustrate the impact of various cooking techniques on the concentration of 5′-nucleotides and their breakdown products. As shown in Figure 4 and Table 4, the levels of IMP, AMP, ADP, hypoxanthine, and total nucleotide in raw meat are much greater than in Pingliang red beef that was cooked. ADP, hypoxanthine, and AMP concentrations in raw beef are reduced by 94%, 84%, and 75%, respectively. The contents of AMP, ADP, and hypoxanthine in cooked Pingliang red beef are extremely low, corroborating the findings of Liu, Xu, and Zhou (2007) [56] that the nucleotide concentrations of ADP and AMP in processed duck meat products are low. The Dunnett test reveals that the total number of nucleotides in cooked Pingliang red beef is considerably lower than in raw meat (*p* < 0.05) (Table 4). Under the same cooking conditions, the total nucleotide concentration in the other three groups, excluding the steamed group, declines as cooking doneness increases, and the IMP content falls as the cooking duration increases (Figure 4). Our findings are congruent with those of Ishiwatari, Fukuoka, Hamada-Sato, and Sakai (2013) [57], who discovered a similar drop in IMP with increasing beef cooking time. According to the results of the two-factor interaction analysis, the four cooking methods have an extremely significant effect on the IMP content (*p* < 0.01), and the different doneness degrees also have an extremely significant effect on the IMP content (*p* < 0.01); the interaction between the two is significant (*p* < 0.01). Some studies have shown that during meat preparation, the thermal breakdown of IMP to inosine is the primary cause of the development of special flavors [19]. As depicted in Figure 4, the concentration of IMP drops with increasing doneness, while the concentration of hypoxanthine decreases with increasing heating duration. During roasting, the concentrations of AMP and hypoxanthine increase, while ADP and hypoxanthine increase with increasing doneness. Despite the sample’s low AMP concentration, AMP and IMP exhibit a synergistic function in creating umami flavor, particularly during roasting and instant boiling. The tendency of AMP and IMP stay unchanged, whereas the content declines as doneness increases. Additionally, according to the results of the two-factor analysis, different cooking doneness degree levels have a significant effect on the AMP content (*p* < 0.05). The behavior of GMP and IMP during roasting and instant boiling is identical. In this study, it was discovered that long heating time and high temperature result in better thermal degradation of nucleotides and, consequently, a better taste. However, when the heating time is too long, the generated heat accelerates the degradation of nucleotides and damages muscle fibers, resulting in nucleotide loss. The synergistic impact of 5′-nucleotide and FAA (Glu, Asp) has the potential to enhance umami flavor considerably [58]. Therefore, we estimated the EUC of Pingliang red beef using 5′-nucleotide and umami amino acids for various cooking techniques. According to Table 4, the EUC is greatest in the boiling group (30 min) (131.05 g MSG/100 g), followed by 100.9 MSG/100 g in the roasting group (10 min), 99.96 MSG/100 g in the instant boiling group, and 36.85 MSG/100 g in the roasting group (40 min). The effect of different cooking methods on EUC values is not significant (*p* > 0.05), but the effect of different cooking doneness degrees is extremely significant (*p* < 0.01), and the interaction effect between the two is significant (*p* < 0.01). Combining Table 4 and Figure 4 demonstrates that for 5′-nucleotide and the umami amino acid Glu, both cooking methods have higher EUC and doneness than the other cooking methods, indicating that the flavor of Pingliang red beef after 30 min of boiling and 10 min of roasting is superior to that of the other cooking methods and doneness.

#### 3.3.3. Free Amino Acids

FAAs considerably contribute to the flavor and aroma of meat via the Maillard reaction and Strecker degradation, which generate volatile chemicals [19]. FAAs contribute sweet, bitter, sour, and umami tastes to foods [29]. As shown in Table 4, the total and majority of the amino acid contents of raw and cooked meat are not significantly different (*p* > 0.05), a finding consistent with that obtained by Greenwood, Kraybill, and Schweigert (1951) who found that the amino acid composition of cooked beef was similar to that of raw meat [59]. The amount of total sweet amino acids is greatest in the cooked (30 min) group and lowest in the raw meat group (Figure 4). The effect of the four cooking methods on total amino acids is not significant (*p* > 0.05), but the effect of different cooking doneness degrees is significant, and the interaction between the two is significant (*p* < 0.01). The total amount of bitter amino acids in raw beef is greater than in cooked Pingliang red beef, and there is no significant difference in the total amino acid content of Pingliang red beef under different cooking methods (*p* > 0.05). This result is consistent with the previous study on nucleotides. As hypoxanthine has a bitter taste, the hypoxanthine level in this study is much higher than that of cooked beef (*p* < 0.05), showing that both bitter nucleotides and bitter amino acids are greatly reduced after cooking in Pingliang red beef (Table 4). This shows that after cooking and processing Pingliang red beef, the raw meat’s bitter taste can be transformed into a fresh and sweet flavor. The overall bitter amino acid content of raw beef is more than that of cooked Pingliang red beef, and there is no significant variation in the total amino acid content of Pingliang red beef under different cooking methods, similar to the earlier work published in *Nucleotides*. The overall amount of sweet amino acids is highest in the boiling (30 min) group and lowest in the raw beef group. Knowledge of the composition of amino acids is essential for distinguishing the distinctive flavor of cooked meat. Aspartic acid and glutamic acid are the amino acids primarily responsible for the umami flavor [21,22]. Glutamate is an essential antecedent to the flavor of cooked beef and plays a crucial function in the development of umami flavor [60]. After cooking, the most important FAA of Pingliang red beef is Glu. Thr, Glu, and Ala were identified as the primary FAAs in five-spice beef by Zou [19]. Hu and Shiau [61] reported that the most important FAA in chicken flesh is Glu, whereas in crab meat, [62] the most important FAA is Glu. Glu was, likewise, found to be the most important flavoring amino acid, which is consistent with the findings of this study and is equivalent to Pingliang red beef under different cooking methods. This study finds no statistically significant variation in aspartic acid levels, while glutamic acid levels are considerably higher after 30 min of boiling (*p* < 0.05) and lowest after 40 min of roasting. Pingliang red beef has a higher moisture content during steaming, boiling, and instant boiling than during roasting, generating a more hydrophilic environment for the beef and stimulating more protein hydrolysis and other water-based processes to generate flavor compounds [60].

#### 3.3.4. Correlation between Non-Volatile Compounds and Umami-Taste Characteristics of Free Amino Acids

**Figure 5:**
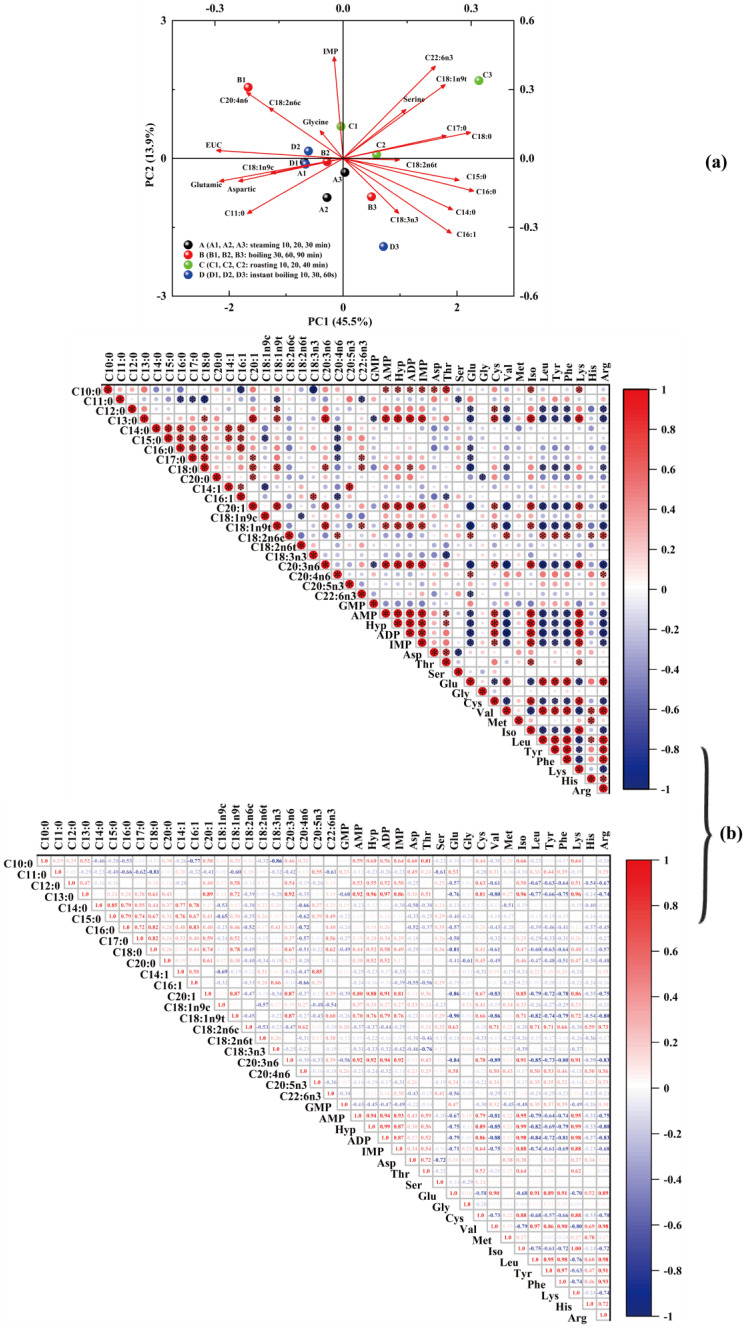
(**a**) Factor loading diagram of two principal components of non-volatile compounds in Pingliang red beef under different cooking methods. (**b**) The relationship between the non-volatile compounds (fatty acids, amino acids, and nucleotides) in Pingliang red beef under different cooking methods.

The differences and correlations between samples were analyzed using correlation and principal component analysis plots to assess the interdependence of non-volatile components (amino acids, fatty acids, and nucleotides) in Pingliang red beef under different cooking methods. Figure 5a depicts the principal component analysis of volatile compounds in Pinliang red beef under various cooking methods and doneness levels. The distribution diagrams of the first two principal components confirmed via PCA are displayed; the total variance contribution rate is 45.5% for the first component and 13.9% for the second. Figure 5b demonstrates that, except GMP, the other four nucleotides (IMP, ADP, AMP, and hypoxanthine) are significantly and positively correlated with each other, indicating that these four nucleotides have a synergistic effect on the flavor of Pingliang red beef under different cooking conditions. Additionally, the signal intensity of pentan-1-ol gradually decreases with the increase in steaming time, and the signal intensity of 1-butanol also gradually decreases with the extension of baking time (Figure 2C). Since IMP and the bitter amino acids tyrosine, phenylalanine, and leucine are significantly negatively correlated, the content of the bitter amino acids tyrosine, phenylalanine, and leucine gradually increases under the same cooking method, whereas the IMP content gradually decreases with longer cooking time; therefore, Pingliang red beef is not suitable for high temperature and long cooking time; moreover, overcooking is not conducive to its flavor. As depicted in Figure 5a, the non-volatile flavor matter of the Pingliang red beef samples differ significantly between cooking methods and doneness levels. To analyze the dependency between free fatty acids, amino acids, and 5′-nucleotides, we employed correlation plots to determine the correlation between the levels of the identified marker metabolites; Figure 5b depicts the Pearson correlation test. Figure 5b demonstrates that IMP, AMP, and ADP are significantly and positively correlated with one another. Similar to the previous results, the correlation coefficients are 0.93 and 0.87, indicating that these three nucleotides have a synergistic effect on the flavor of Pingliang red beef under different cooking conditions. Figure 5b demonstrates that IMP and the bitter amino acids tyrosine, phenylalanine, and leucine are negatively associated, as demonstrated by the correlation coefficients of 0.61, 0.69, and 0.74. In conjunction with the previous study, Pingliang red beef has a higher moisture content during steaming, boiling, and instant boiling than during roasting, generating a more hydrophilic environment for the beef and stimulating more protein hydrolysis and other water-based processes. This makes the volatile flavor substance of Pingliang red beef richer and the taste substance content higher. Additionally, with the increase in doneness degree, the bitter amino acid content increases, the nucleotide content decreases, and the signal intensity of volatile flavor substances decreases, all indicating that Pingliang red beef is not suitable for high temperature and high cooking doneness. The most distinctive feature after 30 min of boiling is the umami, as the highest levels of Glu (*p* < 0.05) and the highest EUC values are obtained with this cooking method.

## 4. Conclusions

In this study, we found that different cooking methods and doneness levels significantly affected the content of volatile and non-volatile compounds (free fatty acids, amino acids, and 5′-nucleotides) in Pingliang red beef, resulting in significant changes in the flavor and taste characteristics of Pingliang red beef. As the cooking process accelerates the oxidation of lipids and the Maillard reaction in Pingliang red beef, it also accelerates the decomposition of fatty acids in meat products; this enriches the flavor substances in the cooked meat product, especially under boiling for 30 min and roasting for 10 min, which promotes the production of aldehydes and alcohols more readily. Notably, roasting for 40 min produces a more bitter flavor than roasting for 20 min; excessive heating can damage the beef muscle fibers, consequently accelerating the thermal breakdown of nucleotides and amino acids, resulting in a loss of nucleotides and amino acids and a decrease in the umami flavor of the cooked meat product. These results could establish a theoretical basis for the flavor and taste alterations of Pingliang red beef under various cooking methods and doneness levels.

## Figures and Tables

**Table 1:** Information on the Pingliang red beef samples and their cooking methods.

IDX	SN	Cooking Method	Internal T (°C)	Total Cooking Time (min)	Details
1	A-1	Steaming for 10 min	About 45 °C	10 min	5 cm × 5 cm × 2.5 cm
2	A-2	Steaming for 30 min	About 75 °C	30 min	5 cm × 5 cm × 2.5 cm
3	A-3	Steaming for 60 min	About 98 °C	60 min	5 cm × 5 cm × 2.5 cm
4	B-1	Boiling for 30 min	About 45 °C	30 min	5 cm × 5 cm × 2.5 cm
5	B-2	Boiling for 60 min	About 75 °C	60 min	5 cm × 5 cm × 2.5 cm
6	B-3	Boiling for 90 min	About 98 °C	90 min	5 cm × 5 cm × 2.5 cm
7	C-1	Roasting for 10 min	About 45 °C	10 min	3 cm × 3 cm × 2.5 cm
8	C-2	Roasting for 20 min	About 75 °C	20 min	3 cm × 5 cm × 2.5 cm
9	C-3	Roasting for 40 min	About 98 °C	40 min	3 cm × 3 cm × 2.5 cm
10	D-1	Instant boiling for 10 s	-	10 s	5 cm × 5 cm × 2 mm
11	D-2	Instant boiling for 30 s	-	30 s	5 cm × 5 cm × 2 mm
12	D-3	Instant boiling for 60 s	-	60 s	5 cm × 5 cm × 2 mm

**Table 2:** Experimental conditions for Pingliang red beef analysis via HS-GC-IMS.

Gas-Phase Ion Mobility Spectrometry Unit	
Analysis time	20 min
Column type	MXT-5 15 m ID: 0.53 mm
Column temperature	60 °C
Carrier gas/drift gas	N_2_
IMS temperature	45 °C
Automatic headspace sampling unit	
Injection volume	500 μL
Incubation time	15 min
Incubation temperature	60 °C
Syringe temperature	65 °C
Incubation speed	500 rpm

**Table 4:** Means of fatty acid, 5′-nucleotide, and free amino acid (mg/100 g^−1^) concentrations in Pingliang red beef under different cooking methods.

Traits	Raw	A-1	A-2	A-3	B-1	B-2	B-3	C-1	C-2	C-3	D-1	D-2	D-3	M ^1^	N ^2^	M × N ^3^
Fatty Acid																
C10:0	0.28 ± 0.1 ^ab^	0.08 ± 0.01 ^bc^	0.06 ± 0.04 ^c^	0.05 ± 0.04 ^c^	0.29 ± 0.16 ^a^	0.15 ± 0.05 ^abc^	0.07 ± 0.004 ^c^	0.06 ± 0.01 ^c^	0.05 ± 0.03 ^c^	0.04 ± 0.002 ^c^	0.1 ± 0.01 ^abc^	0.09 ± 0.06 ^bc^	0.07 ± 0.04 ^c^	**	**	*
C11:0	32.02 ± 1.54 ^d^	49.93 ± 0.93 ^a^	39.8 ± 0.98 ^bc^	34.48 ± 0.98 ^cd^	52.05 ± 1.83 ^a^	53.83 ± 4.33 ^a^	32.81 ± 1.67 ^d^	35.99 ± 0.88 ^cd^	37.37 ± 1.89 ^cd^	15.06 ± 0.03 ^e^	43.23 ± 1.76 ^b^	35.14 ± 0.29 ^cd^	54.62 ± 1.68 ^a^	**	**	**
C12:0	0.07 ± 0.04	0.04 ± 0.02	0.05 ± 0.03	0.06 ± 0.04	0.04 ± 0.01	0.07 ± 0.02	0.03 ± 0.02	0.05 ± 0.01	0.05 ± 0.04	0.05 ± 0.001	0.04 ± 0.02	0.04 ± 0.03	0.04 ± 0.02	NS	NS	NS
C13:0	2.84 ± 1.48 ^a^	0.08 ± 0.04 ^b^	0.08 ± 0.01 ^b^	0.11 ± 0.06 ^b^	0.06 ± 0.04 ^b^	0.05 ± 0.03 ^b^	0.03 ± 0.02 ^b^	0.2 ± 0.02 ^b^	0.32 ± 0.09 ^b^	0.5 ± 0.23 ^b^	0.34 ± 0.22 ^b^	0.41 ± 0.25 ^b^	0.69 ± 0.31 ^b^	**	*	NS
C14:0	1.28 ± 0.22 ^def^	1.1 ± 0.07 ^ef^	1.34 ± 0.06 ^def^	1.74 ± 0.56 ^bcd^	1.05 ± 0.11 ^ef^	1.31 ± 0.07 ^def^	2.1 ± 0.06 ^abc^	1.59 ± 0.07 ^bcde^	1.54 ± 0.04 ^cdef^	2.14 ± 0.01 ^ab^	1.22 ± 0.1 ^def^	0.97 ± 0.06 ^f^	2.57 ± 0.01 ^a^	**	**	**
C15:0	0.36 ± 0.14 ^bc^	0.16 ± 0.05 ^d^	0.26 ± 0.02 ^bcd^	0.28 ± 0.06 ^bcd^	0.22 ± 0.02 ^cd^	0.21 ± 0.01 ^cd^	0.43 ± 0.01 ^b^	0.27 ± 0.06 ^bcd^	0.35 ± 0.03 ^bc^	0.65 ± 0.031 ^a^	0.26 ± 0.1 ^bcd^	0.31 ± 0.01 ^bcd^	0.66 ± 0.05 ^a^	**	**	**
C16:0	16.89 ± 1.42 ^b^	13.45 ± 0.05 ^de^	15.84 ± 0.18 ^bcd^	15.91 ± 0.43 ^bcd^	9.02 ± 0.05 ^f^	12.15 ± 0.22 ^e^	18.1 ± 0.84 ^b^	16.08 ± 1.42 ^bc^	15.98 ± 0.23 ^bcd^	20.94 ± 1.54 ^a^	13.81 ± 0.34 ^cde^	13.11 ± 0.11 ^e^	18.37 ± 0.47 ^b^	**	**	**
C17:0	0.82 ± 0.4 ^ab^	0.32 ± 0.17 ^de^	0.51 ± 0.02 ^bcde^	0.32 ± 0.1 ^de^	0.36 ± 0.04 ^cde^	0.45 ± 0.01 ^bcde^	0.79 ± 0.01 ^bc^	0.76 ± 0.04 ^bc^	0.27 ± 0.01 ^e^	1.19 ± 0.002 ^a^	0.51 ± 0.01 ^bcde^	0.56 ± 0.02 ^bcde^	0.74 ± 0.03 ^bcd^	**	**	**
C18:0	14.19 ± 2.72 ^a^	8.93 ± 0.61 ^bcd^	9.66 ± 0.55 ^bcd^	9.5 ± 0.5 ^bcd^	7.35 ± 0.32 ^d^	7.98 ± 0.19 ^cd^	11.32 ± 0.42 ^b^	11.3 ± 0.38 ^b^	10.75 ± 0.21 ^bc^	15.11 ± 0.03 ^a^	8.82 ± 0.54 ^bcd^	10.49 ± 0.06 ^bc^	10.59 ± 0.09 ^bc^	**	**	**
C20:0	0.14 ± 0.07	0.04 ± 0.02	0.05 ± 0.04	0.09 ± 0.05	0.06 ± 0.03	0.09 ± 0.05	0.16 ± 0.03	0.02 ± 0.01	0.05 ± 0.03	0.05 ± 0.003	0.03 ± 0.02	0.06 ± 0.04	0.1 ± 0.06	**	**	NS
C14:1	0.18 ± 0.07 ^b^	0.19 ± 0.06 ^b^	0.2 ± 0.03 ^b^	0.24 ± 0.13 ^b^	0.24 ± 0.05 ^b^	0.24 ± 0.01 ^b^	0.27 ± 0 ^b^	0.25 ± 0.01 ^b^	0.25 ± 0.04 ^b^	0.31 ± 0.03 ^b^	0.2 ± 0.03 ^b^	0.27 ± 0.06 ^b^	0.58 ± 0.02 ^a^	**	**	**
C16:1	1.78 ± 0.29 ^b^	1.89 ± 0.11 ^b^	2.58 ± 0.54 ^ab^	2.51 ± 0.5 ^ab^	0.89 ± 0.02 ^c^	1.84 ± 0.56 ^b^	2.29 ± 0.09 ^ab^	2.18 ± 0.28 ^ab^	2.24 ± 0 ^ab^	2.68 ± 0.08 ^ab^	1.81 ± 0.03 ^b^	1.94 ± 0.05 ^b^	3.05 ± 0.11 ^a^	*	**	**
C20:1	0.57 ± 0.34 ^a^	0.08 ± 0.02 ^b^	0.1 ± 0.02 ^b^	0.11 ± 0.02 ^b^	0.17 ± 0.06 ^ab^	0.17 ± 0.05 ^ab^	0.26 ± 0.14 ^ab^	0.18 ± 0.06 ^ab^	0.19 ± 0.08 ^ab^	0.29 ± 0.11 ^ab^	0.09 ± 0.02 b	0.17 ± 0.06 ^ab^	0.21 ± 0.1 ^ab^	NS	*	NS
C18:1n9c	26.01 ± 4.5 ^a^	16.99 ± 0.21 ^ab^	22.49 ± 0.64 ^ab^	20.99 ± 0.5 ^ab^	12.07 ± 1.25 ^bc^	16.71 ± 0.37 ^ab^	24.3 ± 0.24 ^ab^	24.83 ± 1.87 ^ab^	12.97 ± 12.94 ^abc^	1.2 ± 1.12 ^c^	18.83 ± 0.85 ^e^	22.29 ± 0.33 ^de^	0.84 ± 0.6 ^c^	*	**	**
C18:1n9t	41.02 ± 7 ^a^	9.16 ± 2.15 ^cde^	10.51 ± 2.53 ^cde^	15.71 ± 3.2 ^cde^	12.97 ± 5.38 ^cde^	14.72 ± 2.74 ^cde^	16.55 ± 4.84 ^cde^	18.9 ± 4.54 ^bcd^	21.01 ± 3.01 ^bc^	30.01 ± 1.34 ^ab^	4.83 ± 2.09 ^bc^	7.45 ± 1.74 ^ab^	8.39 ± 1.6 ^cde^	**	**	*
C18:2n6c	3.32 ± 1.1 ^ef^	7.73 ± 0.7 ^bc^	8.6 ± 0.48 ^abc^	9.62 ± 0.6 ^ab^	12.1 ± 0.37 ^a^	6.87 ± 0.12 ^bcd^	3.64 ± 3.43 ^def^	7.89 ± 0.52 ^bc^	0.33 ± 0.02 ^f^	7.25 ± 0.52 ^bc^	7.48 ± 0.37 ^bc^	10.06 ± 0.34 ^a^	5.42 ± 0.17 ^bc^	**	**	**
C18:2 n6t	1.25 ± 0.16 ^bc^	3.12 ± 2 ^bc^	2.07 ± 0.96 ^bc^	1.23 ± 0.19 ^bc^	1.45 ± 0.42 ^bc^	4.4 ± 1.2 ^b^	0.05 ± 0.01 ^c^	2.71 ± 0.85 ^bc^	9.5 ± 2.32 ^a^	4.46 ± 1.28 ^b^	2.53 ± 0.86 ^b^	1.57 ± 0.9 ^b^	5.19 ± 2.03 ^b^	**	**	**
C18:3n3	1.02 ± 0.15 ^d^	1.38 ± 0.11 ^bc^	1.61 ± 0.07 ^ab^	1.72 ± 0.05 ^a^	0.54 ± 0.01 ^e^	1.34 ± 0.01 ^c^	1.45 ± 0.05 ^bc^	1.56 ± 0.12 ^abc^	1.73 ± 0.01 ^a^	1.4 ± 0.07 ^bc^	1.39 ± 0.03 ^def^	1.78 ± 0.02 ^b^	1.39 ± 0.05 ^g^	**	**	**
C20:3n6	3.04 ± 1.29 ^a^	0.21 ± 0.1 ^b^	0.18 ± 0.09 ^b^	0.15 ± 0.04 ^b^	0.11 ± 0.04 ^b^	0.09 ± 0.02 ^b^	0.04 ± 0.01 ^b^	0.63 ± 0.19 ^b^	0.89 ± 0.31 ^b^	0.95 ± 0.62 ^b^	0.42 ± 0.35 ^b^	0.08 ± 0.01 ^b^	0.08 ± 0.05 ^a^	*	NS	NS
C20:4n6	1.85 ± 0.3 ^ef^	2.63 ± 0.32 ^bcd^	2.55 ± 0.21 ^bcde^	2.94 ± 0.18 ^bc^	4.02 ± 0.32 ^a^	1.97 ± 0.05 ^def^	2.1 ± 0.17 ^def^	2.25 ± 0.22 ^cdef^	2.44 ± 0.16 ^cdef^	1.79 ± 0.172 ^fg^	2.09 ± 0.19 ^def^	3.19 ± 0.08 ^b^	1.18 ± 0.11 ^g^	**	**	**
C20:5	0.27 ± 0.13 ^b^	0.42 ± 0.05 ^b^	0.42 ± 0.04 ^b^	0.48 ± 0.05 ^b^	0.69 ± 0.08 ^b^	0.46 ± 0.05 ^b^	0.28 ± 0.01 ^b^	0.34 ± 0.03 ^b^	0.39 ± 0.05 ^b^	0.16 ± 0.13 ^b^	0.31 ± 0.03 ^b^	0.84 ± 0.03 ^b^	1.84 ± 0.71 ^a^	**	*	**
C22:6n3	0.33 ± 0.21 ^bc^	0.15 ± 0.05 ^bc^	0.11 ± 0.02 ^bc^	0.05 ± 0.03 ^c^	0.3 ± 0.03 ^bc^	0.21 ± 0.1 ^bc^	0.14 ± 0.04 ^bc^	0.18 ± 0.07 ^bc^	0.42 ± 0.03 ^b^	0.92 ± 0.24 ^a^	0.19 ± 0.08 ^bc^	0.12 ± 0.03 ^bc^	0.1 ± 0.07 ^bc^	**	**	**
SFA ^1^	68.88 ± 1.3 ^cde^	74.13 ± 1.96 ^bc^	67.64 ± 0.19 ^def^	62.52 ± 0.26 ^ef^	70.51 ± 1.6 ^bcd^	76.28 ± 4.11 ^b^	65.83 ± 2.9 ^def^	66.3 ± 2.71 ^def^	66.73 ± 1.45 ^def^	55.72 ± 1.75 ^g^	68.35 ± 0.47 ^cde^	61.17 ± 0.23 ^fg^	88.43 ± 1.44 ^a^	**	*	**
MUFA ^2^	69.56 ± 2.6 ^a^	28.29 ± 2.3 ^c^	35.87 ± 2.63 ^bc^	39.57 ± 3.31 ^bc^	26.33 ± 6.75 ^cd^	33.67 ± 3.59 ^bc^	43.66 ± 4.37 ^b^	46.34 ± 2.45 ^b^	36.66 ± 9.89 ^bc^	34.48 ± 2.33 ^bc^	25.76 ± 2.96 ^cd^	32.13 ± 1.24 ^bc^	13.07 ± 1.01 ^d^	**	*	**
PUFA ^3^	11.07 ± 2.36 ^bc^	15.63 ± 3.32 ^ab^	15.53 ± 0.3 ^ab^	16.18 ± 1.06 ^ab^	19.2 ± 0.3 ^a^	15.34 ± 1.19 ^ab^	7.7 ± 3.28 ^c^	15.55 ± 0.16 ^ab^	15.69 ± 2.43 ^ab^	16.93 ± 3.02 ^ab^	14.4 ± 0.31 ^ab^	17.63 ± 1.18 ^ab^	15.18 ± 2.51 ^ab^	NS	*	**
FFAs ^4^	149.5 ± 3.67 ^a^	118.05 ± 7.57 ^bc^	119.04 ± 2.14 ^bc^	118.26 ± 2.51 ^bc^	116.04 ± 5.45 ^bc^	125.29 ± 0.67 ^b^	117.19 ± 4.74 ^bc^	128.19 ± 0.42 ^b^	119.07 ± 6 ^bc^	107.13 ± 2.44 ^c^	108.5 ± 2.8 ^c^	110.92 ± 0.17 ^c^	116.68 ± 4.96 ^bc^	*	*	**
5′-nucleotide																
GMP	0.14 ± 0.03	0.23 ± 0.1	0.32 ± 0.12	0.4 ± 0.12	0.4 ± 0.15	0.4 ± 0.24	0.37 ± 0.21	0.39 ± 0.18	0.26 ± 0.12	0.21 ± 0.06	0.18 ± 0.12	0.19 ± 0.12	0.3 ± 0.12	NS	NS	NS
AMP	0.12 ± 0.03 ^a^	0.03 ± 0.02 ^b^	0.03 ± 0.02 ^b^	0.02 ± 0.01 ^b^	0.03 ± 0.02 ^b^	0.03 ± 0.02 ^b^	0.02 ± 0.01 ^b^	0.05 ± 0.03 ^b^	0.04 ± 0.01 ^b^	0.02 ± 0.01 ^b^	0.03 ± 0.01 ^b^	0.03 ± 0.02 ^b^	0.03 ± 0.02 ^b^	NS	*	NS
Hypoxanthine	0.47 ± 0.08 ^a^	0.02 ± 0.01 ^b^	0.03 ± 0.01 ^b^	0.04 ± 0.01 ^b^	0.02 ± 0.01 ^b^	0.03 ± 0.02 ^b^	0.04 ± 0.01 ^b^	0.03 ± 0.01 ^b^	0.03 ± 0.01 ^b^	0.03 ± 0.02 ^b^	0.02 ± 0.01 ^b^	0.02 ± 0.01 ^b^	0.03 ± 0.01 ^b^	NS	NS	NS
ADP	0.38 ± 0.19 ^a^	0.05 ± 0.01 ^b^	0.06 ± 0.02 ^b^	0.06 ± 0.01 ^b^	0.05 ± 0.02 ^b^	0.06 ± 0.02 ^b^	0.08 ± 0.02 ^b^	0.07 ± 0.03 ^b^	0.08 ± 0.02 ^b^	0.08 ± 0.02 ^b^	0.06 ± 0.02 ^b^	0.05 ± 0.01 ^b^	0.08 ± 0.01 ^b^	NS	NS	NS
IMP	1.01 ± 0.13 ^a^	0.3 ± 0.02 ^cd^	0.13 ± 0.02 ^e^	0.13 ± 0.02 ^e^	0.36 ± 0.04 ^bc^	0.31 ± 0.07 ^bcd^	0.17 ± 0.02 ^de^	0.48 ± 0.02 ^b^	0.35 ± 0.02 ^bc^	0.36 ± 0.01 ^bc^	0.33 ± 0.03 ^bcd^	0.3 ± 0.03 ^cd^	0.16 ± 0.08 ^de^	**	**	**
Total ^5^	2.12 ± 0.33 ^a^	0.63 ± 0.07 ^b^	0.56 ± 0.13 ^b^	0.65 ± 0.15 ^b^	0.85 ± 0.16 ^b^	0.82 ± 0.33 ^b^	0.67 ± 0.26 ^b^	1.02 ± 0.23 ^b^	0.76 ± 0.12 ^b^	0.7 ± 0.05 ^b^	0.61 ± 0.16 ^b^	0.59 ± 0.13 ^b^	0.6 ± 0.17 ^b^	NS	NS	NS
Free amino acid																
Aspartic	0.39 ± 0.11	0.32 ± 0.12	0.34 ± 0.14	0.16 ± 0.1	0.4 ± 0.21	0.27 ± 0.08	0.3 ± 0.09	0.31 ± 0.1	0.26 ± 0.05	0.15 ± 0.05	0.35 ± 0.1	0.37 ± 0.12	0.33 ± 0.12	NS	**	*
Threonine	0.17 ± 0.1	0.13 ± 0.08	0.14 ± 0.1	0.12 ± 0.08	0.17 ± 0.03	0.11 ± 0.03	0.12 ± 0.05	0.13 ± 0.05	0.11 ± 0.03	0.11 ± 0.02	0.14 ± 0.02	0.12 ± 0.01	0.13 ± 0.02	NS	*	NS
Serine	0.14 ± 0.09 ^ab^	0.11 ± 0.07 ^ab^	0.12 ± 0.08 ^ab^	0.36 ± 0.17 ^a^	0.13 ± 0.04 ^ab^	0.09 ± 0.04 ^b^	0.09 ± 0.06 ^b^	0.1 ± 0.07 ^ab^	0.09 ± 0.06 ^ab^	0.34 ± 0.11 ^ab^	0.11 ± 0.04 ^ab^	0.16 ± 0.05 ^ab^	0.1 ± 0.02 ^ab^	NS	**	**
Glutamic	3.56 ± 1.55 ^b^	8.6 ± 1.29 ^ab^	8.81 ± 1.19 ^ab^	9.01 ± 1.3 ^ab^	10.03 ± 1.69 ^a^	7.11 ± 2 ^ab^	7.76 ± 1.78 ^ab^	8.24 ± 2.25 ^ab^	7.06 ± 2.77 ^ab^	5.44 ± 1.35 ^ab^	9.15 ± 2.04 ^ab^	8.98 ± 1.72 ^ab^	8.43 ± 1.22 ^ab^	NS	**	**
EUC	39.18 ± 29.51	86.59 ± 24.05	85.73 ± 18.43	90.15 ± 21.12	131.05 ± 51.32	70.5 ± 42.08	73.54 ± 35.96	100.9 ± 54.57	68.91 ± 47.08	36.85 ± 17.29	99.92 ± 39.81	95.2 ± 35.15	81.34 ± 27.22	NS	**	**
Glycine	0.21 ± 0.1 ^ab^	0.25 ± 0.12 ^ab^	0.19 ± 0.07 ^ab^	0.16 ± 0.06 ^ab^	0.22 ± 0.09 ^ab^	0.14 ± 0.04 ^ab^	0.09 ± 0.05 ^b^	0.77 ± 0.09 ^a^	0.35 ± 0.1 ^ab^	0.15 ± 0.1 ^ab^	0.44 ± 0.15 ^b^	0.25 ± 0.04 ^ab^	0.22 ± 0.06 ^ab^	**	**	**
Cystine	0.25 ± 0.12 ^a^	0.01 ± 0 ^b^	0.09 ± 0.05 ^b^	0.11 ± 0.07 ^b^	0.01 ± 0 ^b^	0.01 ± 0 ^b^	0.01 ± 0 ^b^	0.01 ± 0 ^b^	0.01 ± 0	0.02 ± 0.01 ^b^	0.01 ± 0 ^b^	0.01 ± 0 ^b^	0.01 ± 0 ^b^	*	**	**
Valine	0.06 ± 0.04	0.18 ± 0.04	0.18 ± 0.01	0.18 ± 0.04	0.21 ± 0.07	0.16 ± 0.05	0.16 ± 0.02	0.17 ± 0.02	0.14 ± 0.03	0.16 ± 0.04	0.19 ± 0.07	0.22 ± 0.08	0.18 ± 0.07	NS	NS	NS
Methionine	0.04 ± 0.03 ^ab^	0.02 ± 0.01 ^b^	0.03 ± 0.02 ^ab^	0.03 ± 0.01 ^ab^	0.03 ± 0.02 ^ab^	0.02 ± 0.01 ^b^	0.02 ± 0.01 ^b^	0.02 ± 0.01 ^b^	0.03 ± 0.02 ^ab^	0.02 ± 0.01 ^b^	0.03 ± 0.02 ^ab^	0.07 ± 0.02 ^a^	0.02 ± 0.01 ^b^	NS	**	**
Isoleucine	1.08 ± 0.51 ^a^	0.19 ± 0.02 ^b^	0.19 ± 0.04 ^b^	0.18 ± 0.02 ^b^	0.23 ± 0.07 ^b^	0.15 ± 0.05 ^b^	0.16 ± 0.04 ^b^	0.18 ± 0.06 ^b^	0.15 ± 0.04 ^b^	0.17 ± 0.06 ^b^	0.19 ± 0.08 ^b^	0.21 ± 0.09 ^b^	0.19 ± 0.07 ^b^	NS	**	**
Leucine	0.18 ± 0.06	0.41 ± 0.2	0.43 ± 0.22	0.41 ± 0.2	0.51 ± 0.3	0.34 ± 0.23	0.38 ± 0.17	0.39 ± 0.18	0.33 ± 0.12	0.38 ± 0.07	0.44 ± 0.13	0.45 ± 0.14	0.43 ± 0.12	NS	*	*
Tyrosine	0.07 ± 0.04	0.13 ± 0.07	0.13 ± 0.05	0.12 ± 0.03	0.16 ± 0.05	0.1 ± 0.06	0.11 ± 0.07	0.12 ± 0.03	0.1 ± 0.01	0.11 ± 0.02	0.13 ± 0.04	0.12 ± 0.04	0.13 ± 0.05	NS	NS	NS
Phenylalanine	0.07 ± 0.05	0.14 ± 0.07	0.15 ± 0.09	0.14 ± 0.08	0.18 ± 0.06	0.12 ± 0.06	0.13 ± 0.07	0.14 ± 0.08	0.12 ± 0.06	0.13 ± 0.06	0.16 ± 0.08	0.14 ± 0.06	0.15 ± 0.07	NS	**	**
Lysine	1.98 ± 0.53 ^a^	0.28 ± 0.17 ^b^	0.28 ± 0.14 ^b^	0.28 ± 0.14 ^b^	0.33 ± 0.19 ^b^	0.23 ± 0.09 ^b^	0.25 ± 0.08 ^b^	0.26 ± 0.09 ^b^	0.22 ± 0.08 ^b^	0.25 ± 0.04 ^b^	0.29 ± 0.08 ^b^	0.33 ± 0.09 ^b^	0.28 ± 0.14 ^b^	NS	NS	NS
Histidine	0.09 ± 0.06	0.11 ± 0.08	0.1 ± 0.07	0.1 ± 0.07	0.11 ± 0.08	0.09 ± 0.06	0.1 ± 0.07	0.1 ± 0.07	0.09 ± 0.06	0.1 ± 0.06	0.11 ± 0.07	0.14 ± 0.07	0.1 ± 0.03	*	NS	*
Arginine	0.09 ± 0.04	0.23 ± 0.12	0.23 ± 0.1	0.22 ± 0.09	0.29 ± 0.1	0.19 ± 0.1	0.19 ± 0.1	0.22 ± 0.11	0.18 ± 0.07	0.21 ± 0.09	0.24 ± 0.12	0.28 ± 0.13	0.23 ± 0.12	NS	NS	NS
Umami ^6^	3.95 ± 1.66 ^b^	8.92 ± 1.17 ^ab^	9.14 ± 1.05 ^ab^	9.17 ± 1.2 ^ab^	10.43 ± 1.89 ^a^	7.38 ± 2.08 ^ab^	8.06 ± 1.87 ^ab^	8.54 ± 2.34 ^ab^	7.32 ± 2.82 ^ab^	5.59 ± 1.3 ^ab^	9.5 ± 2.14 ^ab^	9.34 ± 1.83 ^ab^	8.76 ± 1.34 ^ab^	NS	**	**
Bitter ^7^	1.58 ± 0.55	1.27 ± 0.48	1.29 ± 0.41	1.25 ± 0.38	1.55 ± 0.43	1.06 ± 0.16	1.11 ± 0.14	1.19 ± 0.13	1.04 ± 0.09	1.15 ± 0.01	1.33 ± 0.01	1.49 ± 0.05	1.29 ± 0.05	NS	**	**
FAAs ^8^	8.34 ± 2.72	11.09 ± 0.97	11.36 ± 0.88	11.55 ± 0.78	12.99 ± 2.18	9.1 ± 2.31	9.81 ± 2.15	11.09 ± 2.61	9.23 ± 2.82	7.7 ± 1.28	11.93 ± 2.22	11.81 ± 1.58	10.9 ± 1.09	NS	**	**

SFA: saturated fatty acid; MUFA: monounsaturated fatty acid; PUFA: polyunsaturated fatty acid; FFAs: total free fatty acids; total nucleotides: total IMP, AMP, ADP, and hypoxanthine; Umami: sum of aspartic and glutamic acid; Bitter: sum of histidine, arginine, valine, methionine, phenylalanine, isoleucine, and leucine; FAAs: total free amino acids. M ^1^: indicates the significance under different cooking methods; N ^2^: indicates the significance at different doneness degrees. M × N ^3^: indicates the interaction effect between two factors of different cooking methods and different doneness degrees. NS, *, **: non-significant or significant at *p* < 0.05 or *p* < 0.01, respectively. Means followed by different letters within a column are significantly different at *p* < 0.05.

## Data Availability

The data generated from the study are clearly presented and discussed in the manuscript.
